# Genomic Surveillance of ESBL-Producing *Klebsiella pneumoniae* Across Municipal Wastewater and Food Animal Production Environments

**DOI:** 10.3390/pathogens15080820

**Published:** 2026-08-04

**Authors:** Katrina L. Edwards, Deepa Gopal Struble, Jordan C. Deutschlander, Isaiah J. Taylor, Lyndy Harden, Erin Harrell, Mabel Kamweli Aworh

**Affiliations:** 1 Department of Biological and Forensic Sciences, Fayetteville State University, Fayetteville, NC 28301, USA; kedwards7@broncos.uncfsu.edu (K.L.E.); dstruble@broncos.uncfsu.edu (D.G.S.); jordandeutschlander@gmail.com (J.C.D.);; 2 Department of Population Health and Pathobiology, College of Veterinary Medicine, North Carolina State University, Raleigh, NC 27607, USA; lbharden@ncsu.edu (L.H.); eaegan@ncsu.edu (E.H.)

**Keywords:** *Klebsiella* spp., ESBL-producing *Klebsiella*, antimicrobial resistance, wastewater treatment plants, agriculture, poultry, cattle, genomic epidemiology, One Health

## Abstract

Extended-spectrum β-lactamase-producing *Klebsiella pneumoniae* (ESBL-*K. pneumoniae*) is a priority antimicrobial-resistant pathogen with the capacity to persist and disseminate across environmental and food animal production systems. This study conducted genomic surveillance of ESBL-*K. pneumoniae* recovered from municipal wastewater treatment plants and food animal production environments in Fayetteville, North Carolina. A total of 449 wastewater and livestock farm environment samples were analyzed. *K. pneumoniae* was recovered from 162 (36.1%) samples, including 68 (15.1%) ESBL-producing isolates. Antimicrobial susceptibility testing showed that 77.9% of ESBL-producing isolates were multidrug-resistant. Polymerase chain reaction identified *bla_CTX-M-1_*, *bla_SHV_*, and *bla_ACT_* as the predominant resistance genes. Whole-genome sequencing of selected isolates identified diverse antimicrobial resistance determinants, virulence-associated genes, and plasmid replicons, with IncFIB(K) being the predominant plasmid type. Phylogenetic analysis demonstrated close genetic relatedness between wastewater and farm environment isolates, with some differing by only 0–6 single-nucleotide polymorphisms, suggesting possible dissemination between environmental reservoirs. These findings highlight municipal wastewater and food animal production environments as important reservoirs of ESBL-*K. pneumoniae* and reinforce the value of integrated genomic surveillance within a One Health framework to monitor the emergence and spread of antimicrobial resistance.

## 1. Introduction

Antimicrobial resistance (AMR) is recognized as one of the most significant global public health threats of the twenty-first century [1,2]. The increasing prevalence of multidrug-resistant bacterial pathogens has compromised the effectiveness of commonly used antimicrobial therapies and contributed to increased morbidity, mortality, and healthcare costs worldwide [3]. Among Gram-negative bacteria, *Klebsiella* spp., particularly *Klebsiella pneumoniae*, are considered high-priority pathogens because of their capacity to acquire and disseminate multiple AMR determinants [3].

*Klebsiella* spp. are opportunistic pathogens associated with a wide range of infections, including pneumonia, urinary tract infections, bloodstream infections, wound infections, and sepsis [4]. While traditionally associated with healthcare settings, *Klebsiella* spp. have increasingly been detected in environmental reservoirs such as soil, surface water, wastewater, livestock environments, and wildlife populations [5]. Their widespread distribution allows them to act as important vehicles for the dissemination of AMR genes across ecological boundaries [6].

Of particular concern is the emergence of extended-spectrum β-lactamase-producing *Klebsiella* (ESBL-*Klebsiella*) [7,8]. ESBL enzymes hydrolyze third-generation cephalosporins and other β-lactam antibiotics, significantly limiting treatment options and increasing the likelihood of therapeutic failure [8]. ESBL-*Klebsiella pneumoniae* has emerged as a major global public health threat due to its increasing prevalence, broad antimicrobial resistance profiles, and capacity for rapid dissemination across clinical and environmental settings [9,10]. The expansion of ESBL-producing strains—particularly those harboring *bla_CTX-M_*, *bla_SHV_*, and *bla_TEM_* genes—has significantly limited therapeutic options and contributed to rising morbidity and mortality worldwide [11,12]. As a result, ESBL-*K. pneumoniae* is now recognized as a critical-priority pathogen by the World Health Organization and a major driver of AMR in both healthcare and community environments [13,14].

Municipal wastewater and food animal production systems represent important but under-characterized reservoirs of ESBL-producing Enterobacterales [11,15]. Wastewater treatment plants (WWTPs) have emerged as critical environmental reservoirs for antimicrobial-resistant bacteria [11,16,17]. These facilities receive wastewater from residential, industrial, and healthcare sources, creating conditions that promote bacterial interactions and the exchange of resistance determinants [11,16]. Although treatment processes reduce microbial loads, resistant bacteria and resistance genes may persist in treated effluent and subsequently be released into natural environments [18,19].

Similarly, food animal production environments, including livestock feces, manure-amended soils, and farm runoff, provide conditions that support the amplification and dissemination of resistant strains [11,20]. Poultry and cattle operations may contribute to the maintenance and dissemination of resistant bacteria through animal waste, environmental contamination, and direct interactions among animal populations [5,21,22]. The use of antimicrobials in animal husbandry further accelerates the selection of ESBL-producing bacteria, raising concerns about environmental spillover and potential transmission to humans through water, food, and occupational exposure [22,23]. The movement of antimicrobial-resistant bacteria between agricultural environments and wastewater systems represents an important concern within the One Health framework, which recognizes the interconnectedness of human, animal, and environmental health [6,14,24].

Despite growing recognition of the role of environmental reservoirs in AMR transmission, limited information exists regarding the occurrence of ESBL-*Klebsiella* spp. across wastewater and agricultural systems [2,8]. Most existing studies focus on clinical isolates, leaving critical gaps in understanding the diversity, virulence potential, and transmission dynamics of environmental strains [4,9]. In particular, little is known about the genomic relatedness of isolates circulating in wastewater and food animal production systems, the distribution of ESBL genes across these environments, and the role of mobile genetic elements—such as plasmids, integrons, and transposons—in shaping resistance architecture [11,20]. Addressing these gaps is essential for developing a One Health-informed understanding of AMR emergence and spread [6,23,24]. Understanding the prevalence, resistance profiles, and genetic relationships of these isolates is essential for identifying potential transmission pathways and informing mitigation strategies [11,23].

Whole-genome sequencing (WGS) provides a powerful tool for high-resolution surveillance, enabling detailed characterization of resistance determinants, virulence signatures, and phylogenetic relationships across diverse reservoirs [25,26]. Applying WGS to environmental *K. pneumoniae* isolates can reveal hidden transmission pathways, identify shared genetic lineages between wastewater and agricultural sources, and uncover the mechanisms driving persistence and dissemination in complex ecosystems [11,14,16,27].

Therefore, the objective of this study was to investigate the occurrence, AMR characteristics, and genomic relatedness of ESBL-*K. pneumoniae* recovered from municipal wastewater and food animal production environments. Specifically, this study aimed to (i) determine the prevalence of *K. pneumoniae* across environmental sources, (ii) characterize AMR phenotypes and associated resistance genes, and (iii) evaluate the genomic relatedness of isolates using whole-genome sequencing and phylogenetic analysis.

## 2. Materials and Methods

### 2.1. Study Design and Sampling

**Figure 1:**
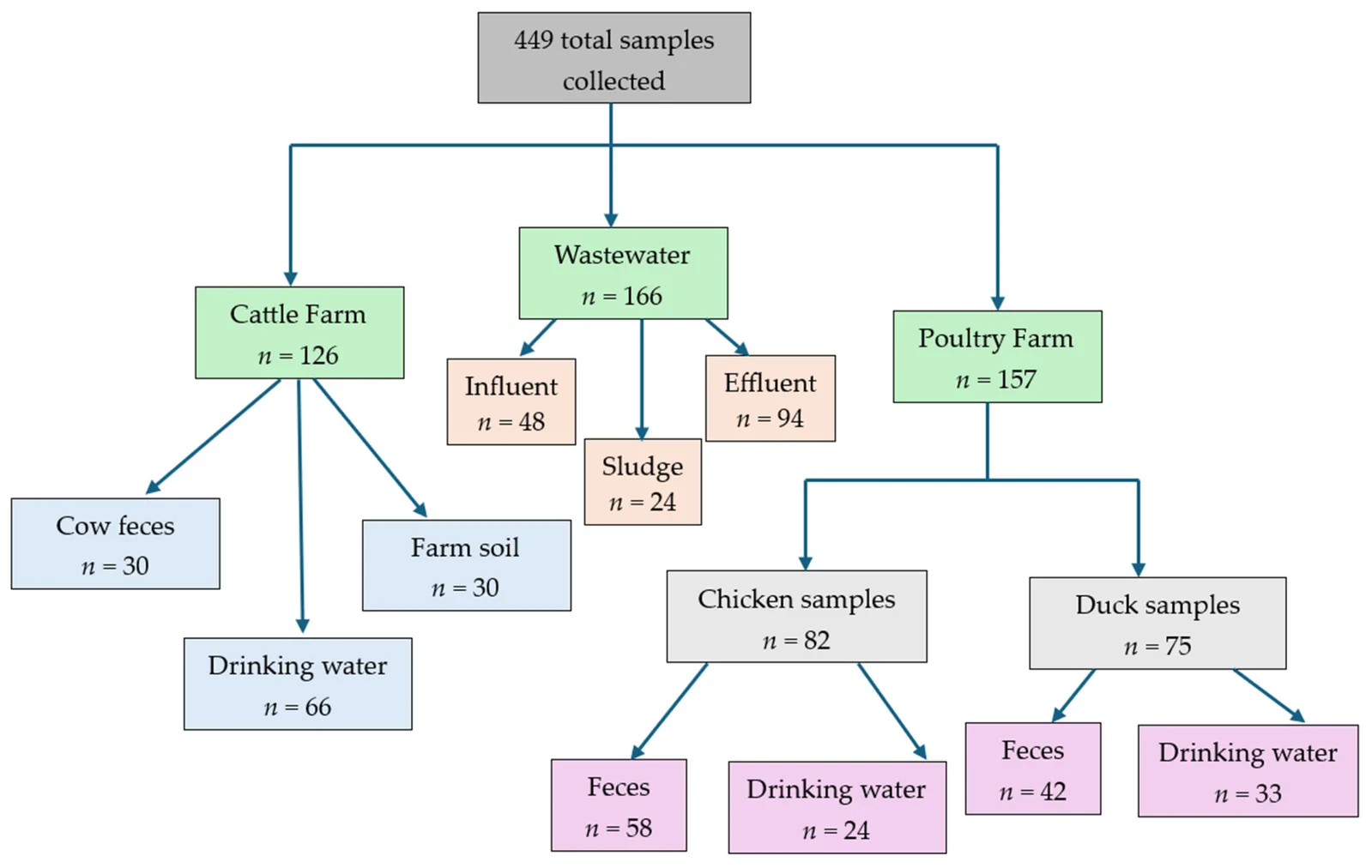
**Schematic representation of sample collection from municipal wastewater and food animal production environments.** The flowchart shows the sampling strategy and distribution of the 449 samples collected from municipal wastewater, cattle farms, and poultry farms. Wastewater samples included influent, effluent, and sludge, while cattle farm samples comprised cow feces, drinking water, and farm soil. Poultry farm samples originating from chickens and ducks included feces and drinking water. Arrows indicate the hierarchical organization of the sampling scheme, linking the total number of samples to each sampling source and its corresponding sample types. Sample sizes for each category are indicated as *n*.

A longitudinal cross-sectional sampling approach was implemented from May to September 2025 to characterize *K. pneumoniae* from environmental and agricultural settings in Fayetteville, North Carolina. Sampling was performed at two municipal wastewater treatment plants and two food production facilities (poultry and cattle farms) to evaluate bacteria populations across interconnected environmental compartments. A total of 449 samples were collected, including wastewater influent, wastewater effluent, activated sludge, animal fecal samples, and farm-associated water sources (Figure 1). All samples were collected aseptically using sterile materials, transported under refrigerated conditions and processed within 3 h of collection to maintain sample integrity.

### 2.2. Isolation and Identification of Klebsiella spp.

Environmental samples were processed for the recovery of presumptive *Klebsiella* spp. using MacConkey agar (Becton, Dickinson and company, Sparks, MD, USA). To enhance the detection of potential ESBL-producing isolates, samples were also cultured on MacConkey agar supplemented with 1 µg /mL cefotaxime (Sigma-Aldrich, St. Louis, MO, USA). Following incubation at 37 °C for 18–24 h, colonies exhibiting typical *Klebsiella* morphology were selected and purified by subculture on Tryptic Soy Agar (Becton, Dickinson and company, Sparks, MD, USA). Species identification was performed using conventional biochemical assays, including oxidase test (Becton, Dickinson and company, Sparks, MD, USA), catalase test (Becton, Dickinson and company, Sparks, MD, USA), Triple Sugar Iron (TSI) agar (Oxoid, Basingstoke, UK), citrate agar (Oxoid, Basingstoke, UK), Lysine Iron Agar (LIA) (Becton, Dickinson and company, Sparks, MD, USA), and Sulfide-Indole-Motility medium (Oxoid, Basingstoke, UK). Confirmed isolates were stored in 15% tryptic soy broth–glycerol at −80 °C until further phenotypic and molecular analyses.

### 2.3. Phenotypic Confirmation of ESBL Production

Extended-spectrum β-lactamase production was confirmed using the Clinical and Laboratory Standards Institute (CLSI) combination disk method [28]. Briefly, ceftazidime and cefotaxime disks were tested alone and in combination with clavulanic acid (Becton, Dickinson and company, Sparks, MD, USA). An increase of at least 5 mm in inhibition zone diameter for either ceftazidime–clavulanic acid or cefotaxime–clavulanic acid compared with the corresponding antibiotic alone was interpreted as confirmation of ESBL production. *Klebsiella pneumoniae* ATCC 700603 and *Escherichia coli* ATCC 25922 were used as ESBL-positive and ESBL-negative quality control strains, respectively.

### 2.4. Antimicrobial Susceptibility Testing

Antimicrobial susceptibility testing was performed using the Kirby–Bauer disk diffusion method according to CLSI guidelines [28]. A 0.5 McFarland standard was used to standardize bacterial inoculum prior to testing. Eight antimicrobial agents representing multiple drug classes were evaluated, including sulfamethoxazole/trimethoprim (1.25/23.75 µg), tetracycline (30 µg), ciprofloxacin (5 µg), cefoxitin (30 µg), ampicillin (10 µg), imipenem (10 µg), chloramphenicol (30 µg), and gentamicin (10 µg) (Becton, Dickinson and company, Sparks, MD, USA).

Briefly, all confirmed ESBL-*K. pneumoniae* isolates stored as stock cultures were first revived by streaking onto Tryptone Soy agar (Becton, Dickinson and company, Sparks, MD, USA) plates prepared according to the manufacturer’s instructions, followed by incubation at 37 °C for 24 h. After incubation, a single colony from each plate was transferred into 2 mL of sterile normal saline using a wooden applicator stick, and the suspension was adjusted to the turbidity of a 0.5 McFarland standard. The standardized inoculum was then spread onto Mueller–Hinton agar (Becton, Dickinson and company, Sparks, MD, USA) plates previously prepared according to manufacturer guidelines. Four antibiotic disks were placed on each plate using sterile forceps, ensuring a spacing of approximately 24 mm between disks. We used *K. pneumoniae* ATCC 700603 as the ESBL-positive control and *Escherichia coli* ATCC 25922 as the ESBL-negative control. The inoculated plates were incubated at 37 °C for 18–24 h, after which inhibition zone diameters were measured and interpreted as susceptible, intermediate, or resistant based on CLSI breakpoints [28]. Multidrug resistance was defined as resistance to three or more antimicrobial classes.

### 2.5. Detection of Antimicrobial Resistance Genes

Genomic DNA was extracted from confirmed *Klebsiella* isolates using a boiling extraction method. The primers used for polymerase chain reaction (PCR) assays were sourced from IDT (Integrated DNA Technologies, Coralville, IA, USA) and are listed in Table 1. PCR amplification was used to detect β-lactamase genes, including *bla_CTX-M-1_* and *bla_SHV_* in multiplex reaction A, *bla_ACT_* and *bla_FOX_* in multiplex reaction B, and *bla_CTX-M-14_* in a singleplex assay performed under the same conditions as multiplex A. Additional multiplex assays targeted aminoglycoside resistance determinants (*aadA1* and *aph(6)*) in multiplex C, as well as quinolone resistance genes (*qnrB*, *qnrS*, and *gyrA*) in multiplex D. For multiplex reactions A-C, each 25 µL PCR mixture consisted of 12.5 µL of Phusion Master Mix, 0.5 µL of each primer, 1 µL of template DNA, and 10.5 µL of nuclease-free water. The amplification protocol for multiplex reactions A and B included an initial denaturation at 95 °C for 5 min, followed by 35 cycles of denaturation at 95 °C for 50 s; annealing at 56 °C or 50 °C, respectively, for 40 s; and a 1-min extension at 72 °C. A final extension step was performed at 72 °C for 5 min. For multiplex reactions D, each 25 µL PCR mixture consisted of 12.5 µL of Phusion Master Mix, 0.5 µL of each primer, 1 µL of template DNA, and 10 µL of nuclease-free water. The amplification protocol for multiplex reactions C and D included an initial denaturation at 95 °C for 5 min, followed by 30 cycles of denaturation at 94 °C for 1 min, annealing at 55 °C for 1 min, and a 2-min extension at 72 °C. A final extension step was performed at 72 °C for 5 min. All PCR reactions were conducted using a SimpliAmpTM Thermal cycler (ThermoFisher Scientific, Waltham, MA, USA). PCR products were separated by electrophoresis on 1% agarose gels stained with SYBR Safe DNA stain (ThermoFisher Scientific, Waltham, MA, USA) and visualized under the Invitrogen iBright FL1500 Imaging System (ThermoFisher Scientific, Waltham, MA, USA). We used a 1 kb DNA ladder (ThermoFisher Scientific, Waltham, MA, USA) as a molecular size marker for this experiment. Positive and negative controls were included in each assay.

### 2.6. Whole-Genome Sequencing

Whole-genome sequencing (WGS) was performed on a subset of 43 of the 68 ESBL-positive *Klebsiella* isolates. Isolates were selected to represent the diversity of the collection with respect to sources of isolation, antimicrobial resistance profiles, and ESBL gene combinations detected by PCR. The selected isolates were intended to capture the major genetic and phenotypic variation present within the ESBL-positive population. The isolates were confirmed as *Klebsiella pneumoniae* with WGS. As previously described, genomic DNA was extracted using a commercial microbial DNA extraction kit (Qiagen, Germantown, MD, USA) according to the manufacturer’s instructions [30]. Extracted DNA was quantified using a Nanodrop spectrophotometer (ThermoFisher Scientific, Waltham, MA, USA), followed by library preparation with standard Illumina kits (Illumina, San Diego, CA, USA).

Sequencing libraries were prepared using Illumina-compatible protocols and sequenced on an Illumina platform to generate paired-end reads as previously described [30]. Briefly, genomic libraries with an average insert size of ~350 bp were generated using the Illumina DNA Prep Kit together with Illumina DNA/RNA UD Indexes (Illumina, San Diego, CA, USA). The indexed libraries were pooled and loaded onto an Illumina NextSeq 1000/2000 P1 Reagent Kit (600-cycle) and sequenced on the NextSeq 2000 platform (Illumina, San Diego, CA, USA) to produce 250-bp paired-end reads. Sequencing quality was evaluated using the MicroRunQC workflow within the GalaxyTrakr environment for GenomeTrakr surveillance (protocols.io) [31]. This pipeline provided quality metrics for raw paired-end FASTQ files, draft de novo assemblies, and multilocus sequence typing (MLST) results for each isolate. Summary outputs included read quality indicators, contig counts, estimated genome size, sequence type (ST), and additional assembly statistics such as median insert size, mean read length, and N50 values.

### 2.7. Bioinformatic and Phylogenetic Analyses

Genome assembly was performed using SPAdes v4.2.0 [32] after quality control processing of the sequencing data. The resulting assemblies were annotated with Prokka v1.14.0 [33] to identify coding sequences and genomic features. AMR genes and plasmid replicons were identified using publicly available databases, including Resfinder v4.7.2 and Plasmidfinder v3.0.3, respectively [34,35]. To investigate the genomic context of AMR and virulence determinants, genome assemblies were analyzed using MobileElementFinder v1.0.3 (Center for Genomic Epidemiology, CGE) with MobileElementFinder database v1.0.2 [36]. The analysis identified mobile genetic elements (MGEs) and determined their association with plasmid replicons, AMR genes, and virulence-associated genes using the default thresholds (≥90% nucleotide identity and ≥60% sequence coverage).

To investigate genomic relatedness, a pan-genome was reconstructed with Panaroo v1.5.1, from which the shared core genome alignment was derived [37]. Variable nucleotide positions within the core genome were identified using SNP-sites v2.5.1 [38]. Genetic distances between isolates were determined by calculating pairwise single-nucleotide polymorphism (SNP) differences with Snp-dists v0.8.2 [39].

The core genome alignment was subsequently used to infer a maximum-likelihood phylogeny in RAxML v8.2.13 (raxmlHPC) under the GTRGAMMA substitution model, using a parsimony-derived starting tree and a random seed of 12345 [40]. Briefly, all 43 isolates were included in the pangenome analysis, but only 39 met the criteria for inclusion in the final core genome alignment used for SNP-based phylogenetic reconstruction. Four isolates were excluded due to insufficient core genome representation.

To evaluate the genetic relationships among isolates from wastewater and food production environment sources, a maximum-likelihood phylogenetic tree was constructed and visualized using iTOL v7 [41]. All sequenced isolates were submitted to the NCBI database, where they were assigned unique Sequence Read Archive (SRA) and BioSample accession numbers under the umbrella BioProject PRJNA1092662 (Supplementary Table S1).

### 2.8. Statistical Analysis

Descriptive statistics were applied to summarize the prevalence of isolates, AMR profiles, resistance gene distributions, and plasmid types. Differences in the frequencies of resistance genes among the various sample sources were assessed using Fisher’s exact test. Differences in the number of plasmid replicons and AMR genes per isolate among sampling locations were assessed using the Kruskal–Wallis rank-sum test. When the overall test was significant, pairwise comparisons were performed using Wilcoxon rank-sum tests with Benjamini–Hochberg correction to control for multiple testing. Differences in the prevalence of individual plasmid replicons and AMR genes among locations were evaluated using Pearson’s chi-square test, and *p*-values were adjusted for multiple comparisons using the Benjamini–Hochberg false discovery rate (FDR) method. Statistical analyses were performed in R v.4.4.1, and a two-sided *p*-value < 0.05 was considered statistically significant [42].

## 3. Results

### 3.1. Prevalence of K. pneumoniae in Wastewater and Food Production Environments

**Figure 2:**
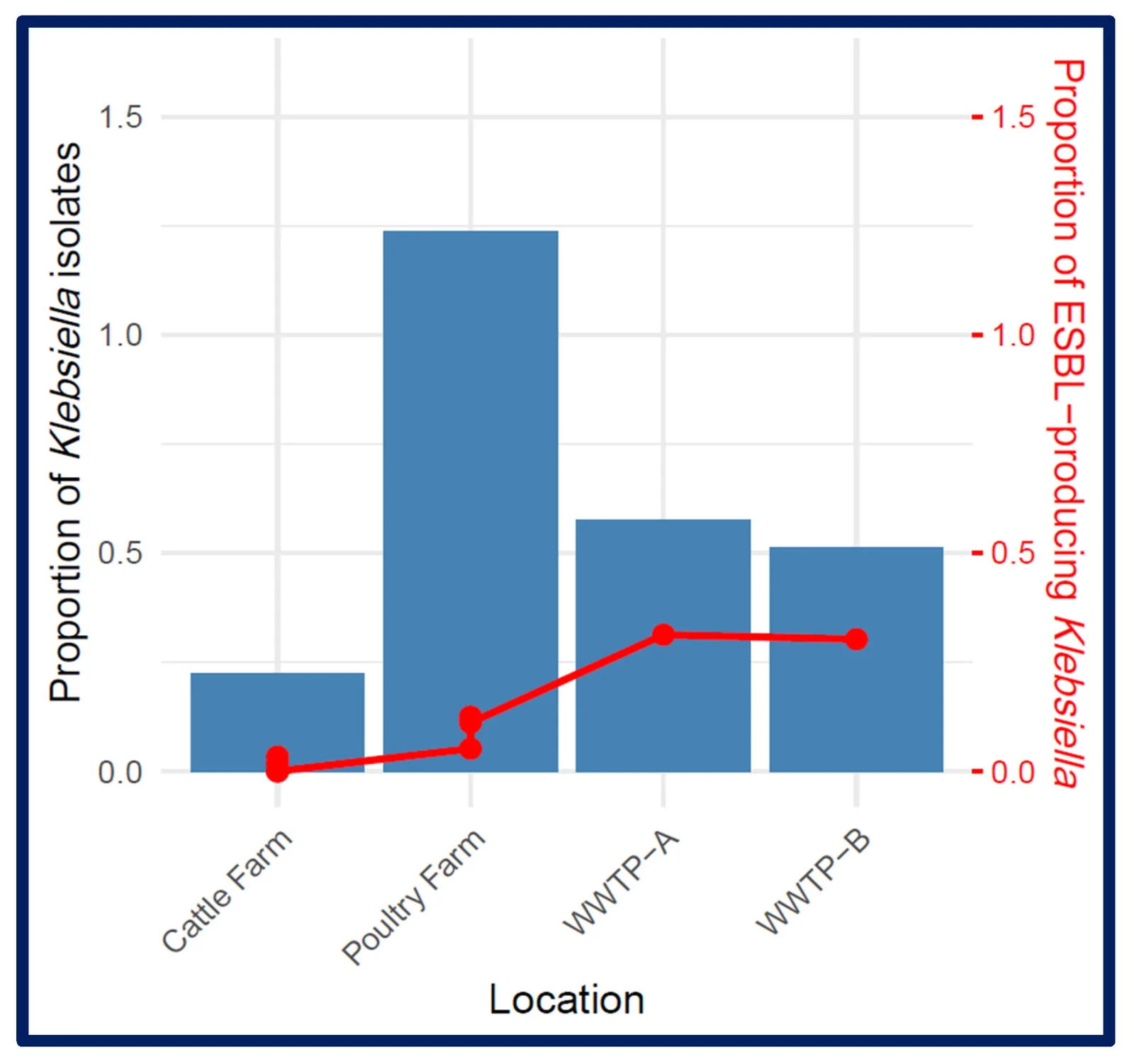
**Proportion of ESBL-producing *Klebsiella* spp. detected among all *Klebsiella*-positive isolates collected from municipal wastewater treatment plants and food production environments (poultry farms and cattle farms).** The figure illustrates the relative contribution of each sampling source to the total number of ESBL-positive isolates. Bars represent the total number of *Klebsiella* isolates recovered from each sampling location (primary y-axis). The red line represents the proportion (%) of ESBL-producing *Klebsiella* isolates among the total *Klebsiella* isolates for each sampling location (secondary y-axis).

The study analyzed a total of 449 samples across municipal and food production environments in North Carolina to determine the presence of ESBL-*K. pneumoniae*. Out of 449 samples analyzed, 162 (36.1%) were presumptively identified as Klebsiella spp. based on colony morphology and biochemical characterization, including oxidase, catalase, citrate utilization, indole production, triple sugar iron (TSI), lysine decarboxylase, and sulfide-indole-motility (SIM) tests. Of these, 68 (15.1%) were confirmed as ESBL-producing *Klebsiella* isolates (Figure 2).

The occurrence of ESBL-*K. pneumoniae*-positive isolates differed across sampling environments. Municipal WWTPs contributed the largest proportion, accounting for 76.5% (52/68) of all ESBL-*K. pneumoniae* recovered, with 26 isolates from WWTP-A and 25 from WWTP-B. Poultry farm environment samples yielded 22.1% (15/68) of the isolates, while cattle farm samples contributed the remaining two isolates. Within the wastewater system, influent (*n* = 31) and activated sludge (*n* = 12) samples produced the highest number of positive detections.

### 3.2. Antimicrobial Susceptibility and Resistance Profiles

Among ESBL-*Klebsiella* isolates, 77.9% (53/68) were multidrug-resistant. The majority of MDR isolates were recovered from wastewater samples at WWTP-A (39.6%, 21/53), followed by WWTP-B (32.1%, 17/53) and poultry farms (24.5%, 13/53). The lowest proportion was observed among isolates from cattle farms (2.8%, 2/53). Antibiotic resistance among ESBL-*Klebsiella* isolates was highest to ampicillin (83.8%, 57/68), ciprofloxacin (57.4%, 39/68), trimethoprim/sulfamethoxazole (52.9%, 36/68) and cefoxitin (52.9%, 36/68), as shown in Table 2.

### 3.3. Molecular Characterization of Resistance Genes Using PCR

**Figure 3:**
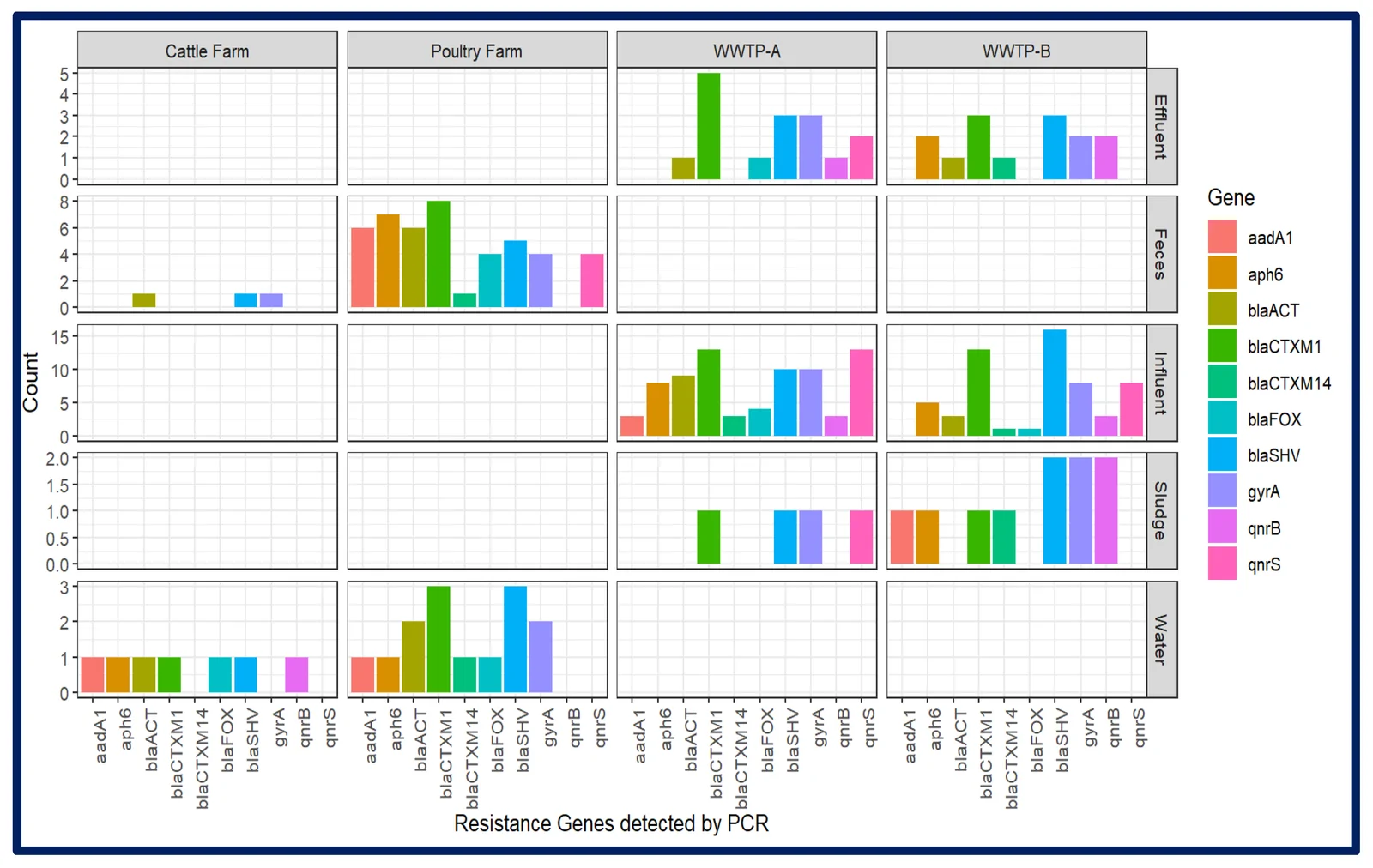
**Distribution of antimicrobial resistance genes detected in *Klebsiella* spp. isolated from municipal wastewater treatment plants and food production environments.** Bar heights represent the number of isolates carrying each resistance gene. Columns correspond to sampling locations (cattle farm, poultry farm, WWTP-A, and WWTP-B), while rows represent sample types (effluent, feces, influent, sludge, and water). Colors denote individual resistance genes. The figure illustrates variation in the prevalence and diversity of antimicrobial resistance determinants across sampling locations and sample types, with wastewater-associated isolates generally harboring a broader range of resistance genes than farm-associated isolates.

PCR screening of the 68 ESBL-*Klebsiella* isolates revealed the presence of multiple ESBL genes, including *bla_CTX-M-1_* (48/68), *bla_SHV_* (45/68), and *bla_CTX-M-14_* (8/68), and AmpC genes including *bla_ACT_* (24/68) and *bla_FOX_* (12/68). Other resistance genes included *aadA1* (12/68), *aphA6* (25/48), *qnrB* (12/68), *qnrS* (28/68) and *gyrA* (33/68). In food production environments, most of these genes were detected in samples originating from poultry operations when compared to cattle farms (Figure 3). The highest frequency of *bla_CTX-M-1_* was observed in WWTP-A isolates (19/26), followed by WWTP-B isolates (17/25) and poultry farm isolates (11/15), with lower levels detected in samples from cattle farms (1/2).

### 3.4. WGS-Based Detection of Resistance Genes

**Figure 4:**
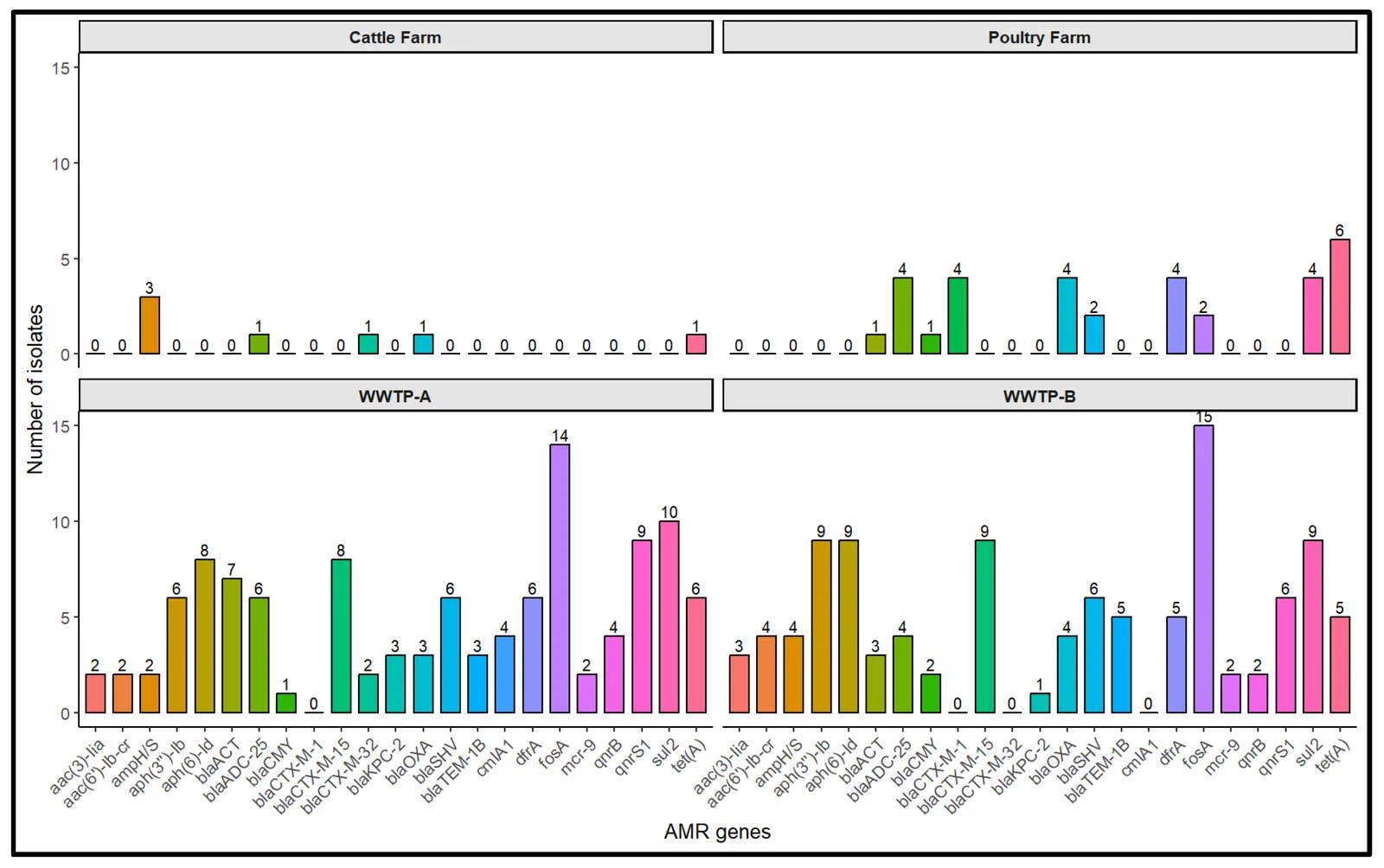
**Distribution of Antimicrobial Resistance Genes Across Sampling Locations.** This figure shows the frequency of antimicrobial resistance (AMR) genes detected in *Klebsiella* isolates collected from different sampling locations. Each facet represents a single location, and the height of each bar corresponds to the number of isolates carrying a specific resistance gene. Bars are labeled with their respective counts to highlight variation in AMR gene prevalence across sites.

Genomic sequencing identified 65 distinct AMR genes among the 43 *K. pneumoniae* isolates analyzed. The most prevalent ESBL gene was *bla_CTX-M-15_*, detected in 17 isolates (39.5%), followed by *bla_ADC-25_* (AmpC β-lactamase) in 15 isolates (34.9%). Carbapenemase genes were less common, present in four isolates (9.3%). The most frequently detected resistance genes are summarized in Figure 4. A wide range of β-lactamase variants was identified, including *bla_ACT-16_*, *bla_CTX-M-1_*, *bla_CTX-M-15_*, *bla_CTX-M-32_*, *bla_OKP-B_*, *bla_SHV-106_*, *bla_SHV-27_*, *bla_ADC-25_*, *bla_CEPH-A3_*, *bla_CMY_*, *bla_MOX_*, *bla_TEM-1B_*, *bla_OXA-1_*, *bla_OXA-270_*, *bla_OXA-304_*, *bla_OXA-98_*, and *bla_KPC-2_*. Among these, the CTX-M family represented the classical ESBL markers. The AmpC-type β-lactamase gene *bla**_CMY_*** was detected in two variants: *bla_CMY-8b_* and *bla_CMY-109_*.

Aminoglycoside resistance genes were the most diverse, with eight variants identified: *aadA2*, *aac(3)-IIa*, *aac(6’)-Ib-cr*, *aph(3’)-Ia*, *aph(3*”*)-Ib*, *aph(6)-Id*, *ant(2’)-Ia*, and *ant(3*”*)-Ia*. Three ESBL-*Klebsiella* isolates carried *aac(6’)-Ib-cr*, which reduces ciprofloxacin susceptibility. The *ant(3*”*)-Ia* gene encoding an aminoglycoside-modifying nucleotidyltransferase was detected in one isolate from a poultry farm. Resistance to folate pathway inhibitors was represented by six genes (*sul1*, *sul2*, *sul3*, *dfrA1*, *dfrA12*, and *dfrA14*). Quinolone resistance genes, classified by the WHO as critically important due to their clinical relevance, were found in ten variants, including *aac(6’)-Ib-cr*, *qnrB1*, *qnrB19*, *qnrB38*, *qnrE1*, *qnrS1*, *qnrS2*, *qnrVC4*, *oqxA*, and *oqxB*. Additional AMR determinants included phenicol resistance genes (*cmlA1*, *floR*), fosfomycin resistance genes (*fosA*, *fosA6*, *fosA7*), tetracycline resistance genes (*tetA*), carbapenemase genes (*cphA4*, *cphA6*), and colistin resistance genes (*mcr-7*, *mcr-9*).

Of the 43 ESBL-*Klebsiella* isolates selected for WGS, carbapenemase genes were detected in four isolates. The carbapenemase genes identified were *cphA4*, *cphA6*, and *blaKPC-2.* Two of the four carbapenemase gene-positive isolates (50%) were found to be phenotypically resistant to imipenem by disk diffusion testing. Details regarding isolate source, sample type, carbapenemase gene content, and imipenem susceptibility phenotype can be seen in Table 3.

### 3.5. Plasmid Analysis and Genetic Mobility

**Figure 5:**
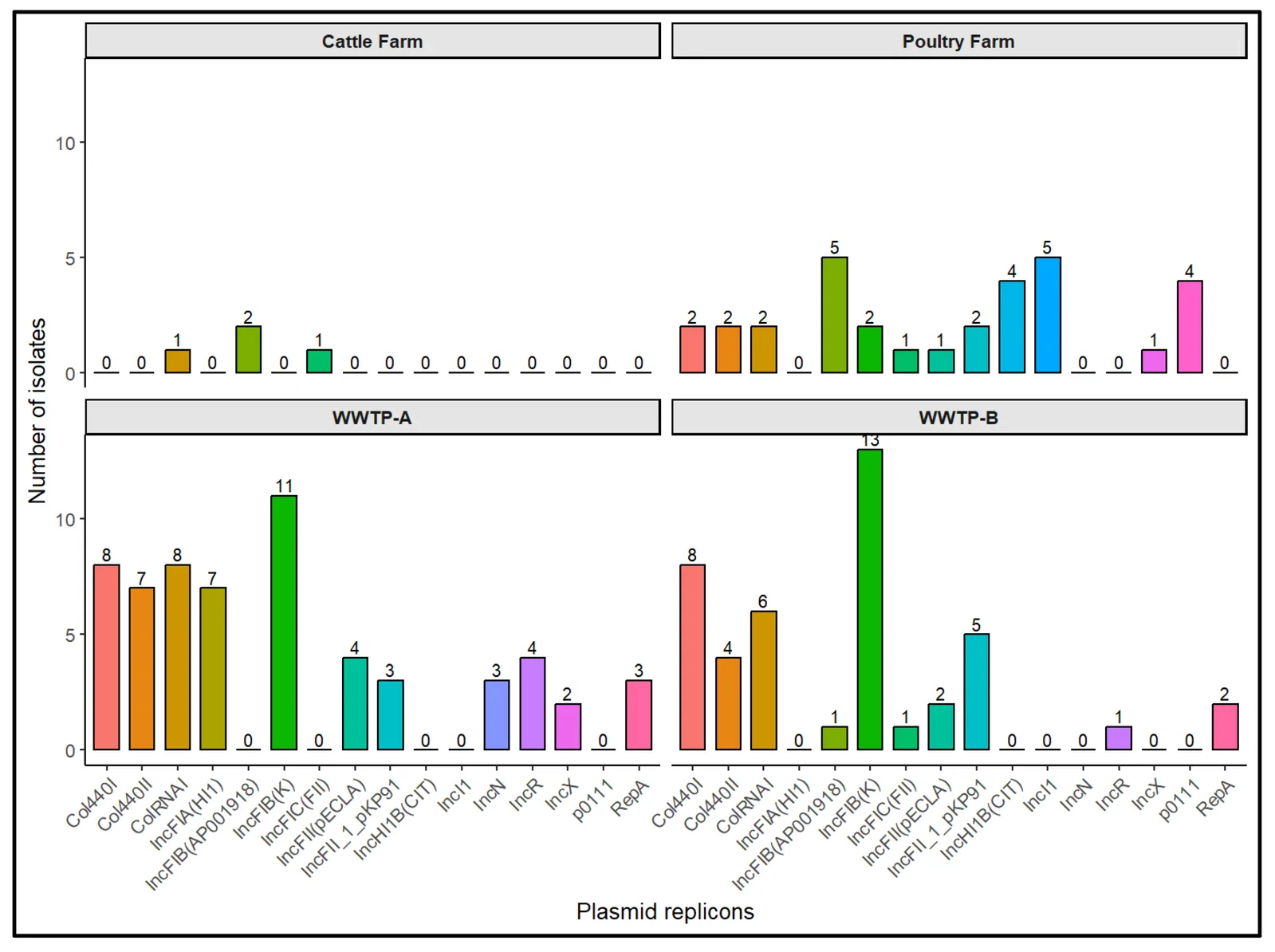
**Plasmid carriage among municipal wastewater and food production environment *Klebsiella* spp. isolates.** This figure illustrates the distribution and diversity of plasmid replicon types detected in *Klebsiella* isolates collected from municipal wastewater treatment plants and farm environments. The bars illustrate the number of isolates carrying each plasmid replicon type, allowing comparison of plasmid prevalence across different sampling locations. The wide diversity of plasmid families observed highlights the potential for horizontal gene transfer and underscores their role in the spread of antimicrobial resistance within and between environmental and agricultural settings.

Thirty (30) different plasmid replicon types were detected among 43 *K. pneumoniae* isolates; however, 11.6% (5/43) did not harbour any plasmid replicons. The most prevalent plasmid replicons detected in descending order were IncFIB(K) (26/43); Col440I (18/43); ColRNAI (17/43); Col440II (13/43); IncFII-1-pKp91 (10/43); IncFIB(AP001918) (8/43); IncFIA (HI1) 7/43); IncFII(pECLA) (7/43); IncI1 (5/43); IncR (5/43); and RepA (5/43). Plasmid content varied among the sequenced isolates, with the greatest diversity observed in wastewater-derived isolates (Figure 5). IncFIB(K), Col440I, and IncFII-1KpP91 were the predominant plasmid replicons identified in duck water isolates, whereas IncFIB(AP001918) was the most frequently detected plasmid replicon among cattle farm-derived isolates. Three isolates exhibited the highest plasmid burden, each carrying 9 of the 30 plasmid replicon types identified in this study. These included one isolate from duck drinking water and two isolates from WWTP-A (one influent and one effluent sample).

### 3.6. Differences in AMR and Plasmid Burden by Location

**Figure 6:**
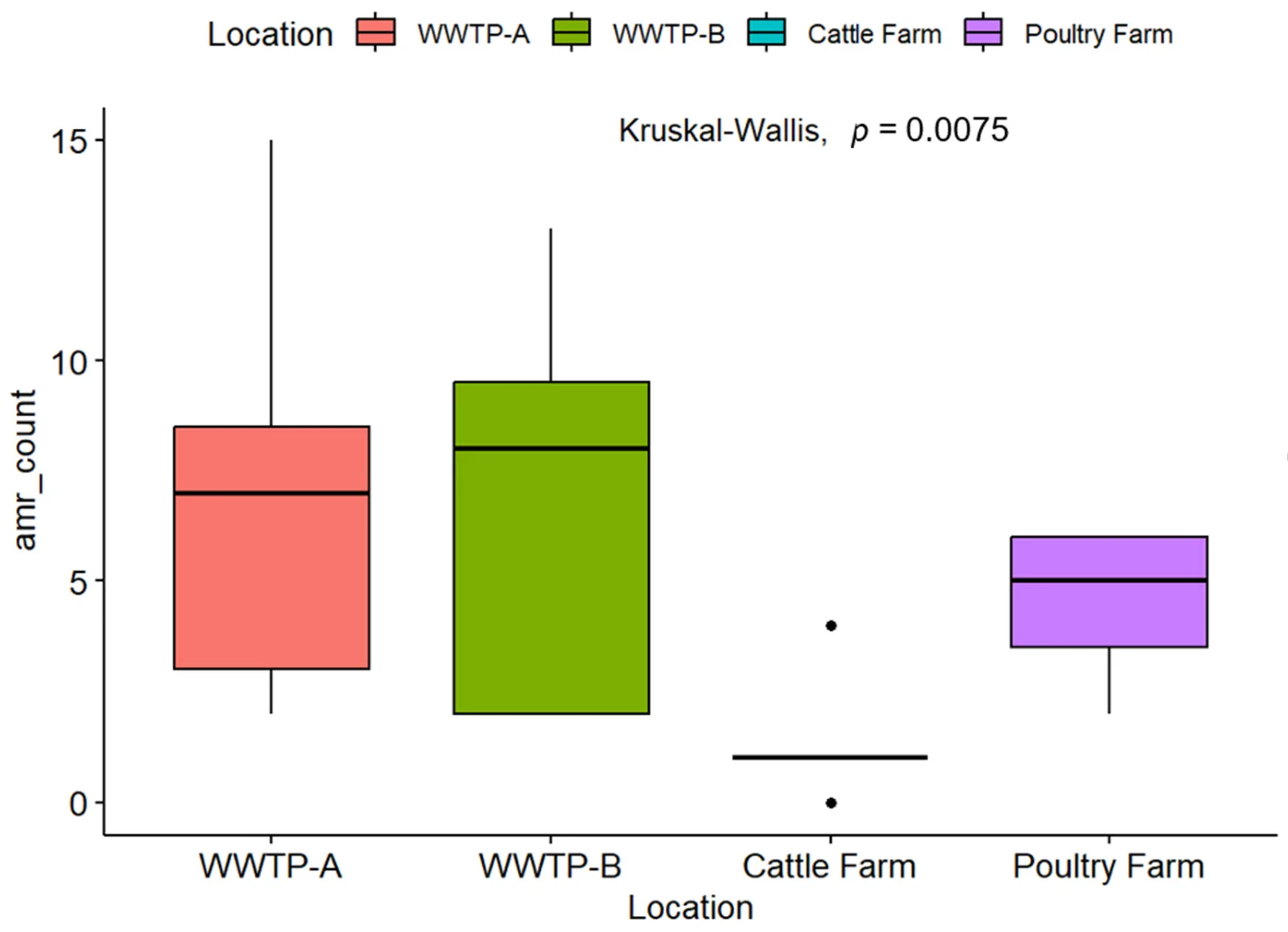
**Distribution of resistance gene counts across sampling locations.** AMR gene burden (number of resistance genes per isolate) varied across sampling locations. Boxplots display the median (central line), interquartile range (box), and 1.5× interquartile range (whiskers), with individual dots representing outliers. The cattle farm environment isolates show no visible box because the interquartile range was zero due to low variability and small sample size; only the median line and two individual observations are displayed. Overall differences among groups were assessed using the Kruskal–Wallis rank-sum test (*p* = 0.0075), indicating a statistically significant variation in AMR gene counts across locations.

The number of AMR genes per isolate varied significantly across locations, as shown in Figure 6 (Kruskal–Wallis χ^2^ = 11.96, df = 3, *p* = 0.0075). Overall, isolates from WWTP-B (median = 8, IQR = 7.5) and WWTP-A (median = 7, IQR = 5.5) exhibited higher AMR gene burdens compared with poultry farm isolates (median = 5, IQR = 2.5) and cattle farm isolates (median = 1, IQR = 0). Post hoc pairwise Wilcoxon rank-sum tests with Benjamini–Hochberg correction showed that cattle farm isolates differed significantly from poultry farm (adjusted *p* = 0.033), WWTP-A (adjusted *p* = 0.014), and WWTP-B (adjusted *p* = 0.014) isolates. No significant differences were observed between poultry farm and WWTP-A (adjusted *p* = 0.197), poultry farm and WWTP-B (adjusted *p* = 0.197), or WWTP-A and WWTP-B (adjusted *p* = 0.889). These results indicate a gradient of increasing AMR gene burden from cattle farm isolates to wastewater-associated isolates. However, these comparisons should be interpreted with caution because the sample sizes were unbalanced across locations, particularly for cattle farm isolates (*n* = 2). Despite this limitation, the data suggests a trend toward higher AMR gene burdens in wastewater-associated isolates than in farm-associated isolates.

**Figure 7:**
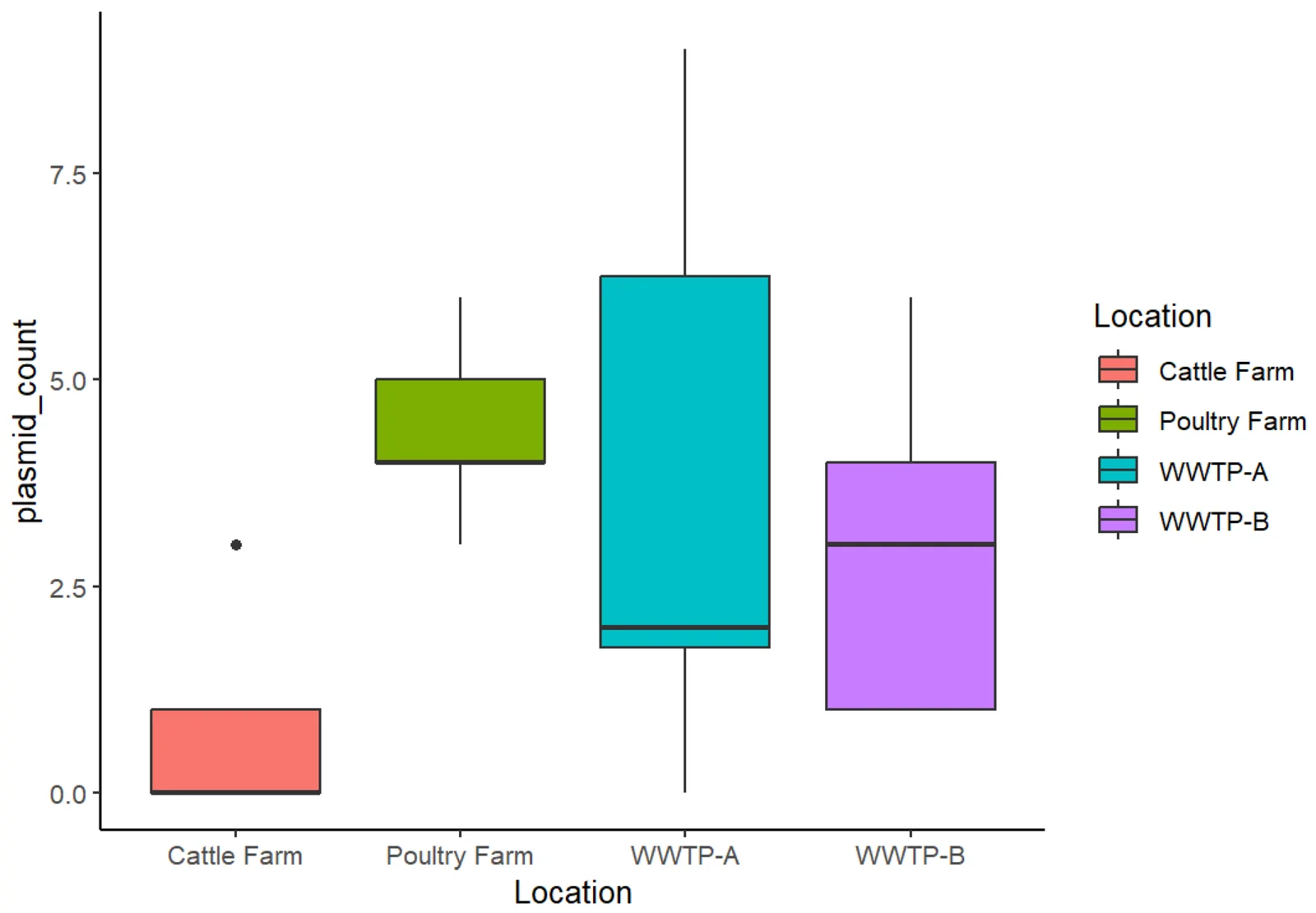
**Distribution of plasmid replicon counts per isolate across sampling locations.** Plasmid replicon burden (number of plasmid incompatibility groups per isolate) varied across sampling locations. Boxplots show the median (central line), interquartile range (box), and whiskers representing 1.5× the interquartile range, with individual dot indicating an outlier. Dots indicate outlier observations beyond 1.5× the interquartile range. Overall differences among locations were statistically significant based on the Kruskal–Wallis rank-sum test (χ^2^ = 9.76, df = 3, *p* = 0.021), indicating unequal plasmid burdens across ecological sources.

The number of plasmid replicons per isolate differed significantly among sampling locations (Kruskal–Wallis χ^2^ = 9.76, df = 3, *p* = 0.021), indicating unequal plasmid burden across sites, as shown in the plasmid boxplot (Figure 7). This suggests location-associated differences in plasmid acquisition or maintenance in *Klebsiella*. Post hoc tests are required to identify the specific groups driving these differences. At the replicon level, several plasmid types varied significantly by location after FDR correction, including IncI1, IncFIB(AP001918), IncHI1B(CIT), p0111, IncFIB(K), and IncFIA(HI1) (FDR < 0.05), whereas others showed no significant differences, indicating selective, rather than uniform, spatial distribution of plasmid incompatibility groups.

### 3.7. Distribution of Plasmid Replicons Carrying AMR and Virulence Genes

Among the sequenced *K. pneumoniae* isolates, five different plasmid replicons were identified carrying AMR genes (Table 4). In addition to AMR-associated plasmids, several isolates also harbored plasmids carrying virulence-associated genes and/or mobile genetic elements (MGEs). Notably, a cattle farm water isolate carried two virulence-associated plasmids: IncFII(pSE11) encoding *traT* and IncFIB(AP001918) encoding *ompT*. Multiple MGEs were detected across different contigs in this isolate, including ISEc1, ISEsa1, ISEc31, IS609, and MITEEc1. IS609 was co-located on the same contig as the virulence gene *hlyE*, suggesting a potential role in its mobilization or genetic linkage. In addition, ten *K. pneumoniae* isolates carried *bla_CTX-M-1_* associated with the insertion sequence ISEc9, indicating potential IS-mediated dissemination of this ESBL gene.

### 3.8. Phylogenetic Analysis and Genomic Relatedness of K. pneumoniae Isolates

**Figure 8:**
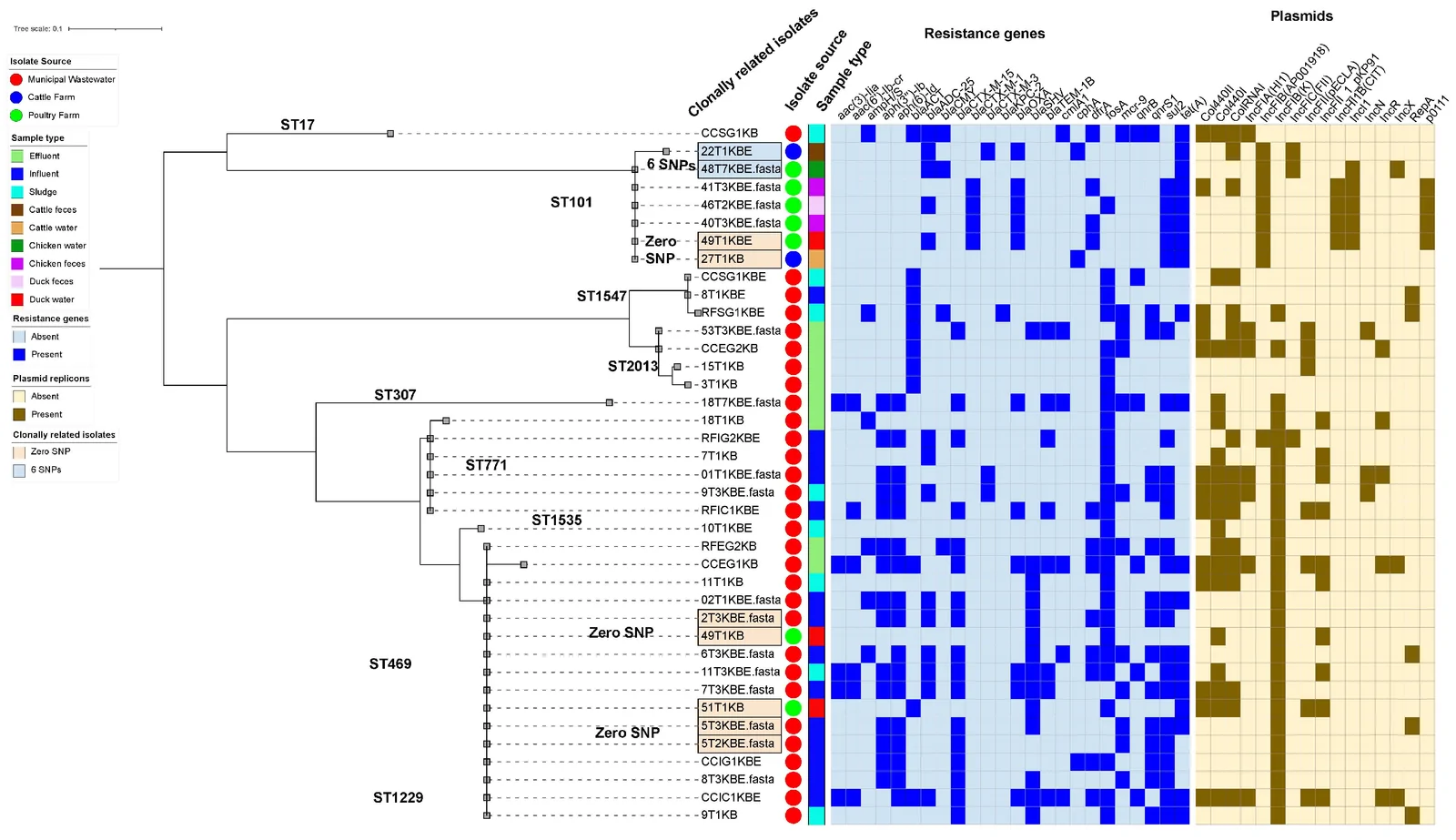
**SNP-based maximum-likelihood phylogeny of ESBL-producing *Klebsiella pneumoniae* isolates recovered from municipal wastewater and food animal production environments.** The phylogenetic tree was reconstructed from a core genome SNP alignment generated through pan-genome analysis. Metadata displayed alongside the tree include isolate source, sample type, antimicrobial resistance (AMR) genes, virulence-associated genes, and plasmid replicons. AMR genes are shown in blue, and plasmid replicons in brown. Isolates are annotated according to their environmental source (wastewater treatment plant or livestock farm) and sample type to facilitate comparison of genomic relatedness across sampling environments. Gray squares indicate branch nodes. Solid lines represent the primary phylogenetic branches, while dashed lines connect terminal branches to isolate labels to improve readability and facilitate isolate identification.

Overall, 39 of the 43 sequenced isolates were included in the core genome single-nucleotide polymorphism (SNP)-based maximum-likelihood phylogenetic analysis (Figure 8). The *K. pneumoniae* isolates recovered from municipal wastewater and food animal production environments exhibited considerable genomic diversity, with most isolates forming distinct phylogenetic clusters. MLST analysis revealed that ST101, ST771, ST469, ST1229, ST17, and ST1547 were the predominant sequence types (Figure 8). Isolates belonging to the same ST generally clustered together on the phylogenetic tree, consistent with their genetic relatedness. Close genetic relatedness was observed among a small number of isolates. Notably, two wastewater influent isolates and one duck farm drinking water isolate belonging to ST469 clustered together on the tree and were genetically indistinguishable, with 0 core genome SNP differences. In addition, one duck drinking water isolate (ST101) clustered with a cattle drinking water isolate (ST101), and a chicken drinking water isolate (ST101) was separated from a cattle fecal isolate (ST101) by 6 core genome SNPs (Figure 8).

## 4. Discussion

This study provides a One Health genomic assessment of ESBL-producing *K. pneumoniae* recovered from municipal WWTPs and food animal production environments in southeastern North Carolina. The findings demonstrate that these environments harbor genetically diverse *K. pneumoniae* populations carrying a broad range of AMR determinants, virulence-associated genes, and plasmid replicons. The detection of ESBL, AmpC, and carbapenemase-associated genes, together with plasmid-mediated resistance mechanisms, underscores the capacity of environmental *K. pneumoniae* populations to maintain clinically relevant resistance profiles outside of healthcare settings [43,44,45]. In addition, the presence of MGEs and diverse plasmid types highlights the role of horizontal gene transfer in shaping the genomic architecture of these isolates [46,47,48].

The observed distribution of ESBL-*K. pneumoniae* across sampling environments in the present study highlights wastewater treatment systems as major points of concentration for these organisms and aligns with findings reported in previous studies [16,49]. The higher detection rate in municipal wastewater compared to food animal production environments suggests that wastewater systems may integrate diverse sources of antimicrobial-resistant bacteria, including human, industrial, and environmental inputs, thereby increasing the likelihood of recovery [50,51,52]. The predominance of isolates in influent and sludge further indicates that both incoming waste streams and treatment-associated solids may serve as key compartments for bacterial persistence and enrichment within treatment facilities [16,52]. In contrast, the lower recovery from livestock environments, particularly cattle farms, may reflect differences in antimicrobial exposure, host density, environmental persistence, or sampling heterogeneity [49,50]. The detection of ESBL-*K. pneumoniae* across both wastewater and agricultural settings nonetheless underscores the ecological connectivity between these systems [50]. Our study findings support the concept that wastewater treatment plants may function as important reservoirs and mixing points for antimicrobial-resistant *K. pneumoniae*, with potential implications for environmental dissemination and consistent with the reports of previous studies [16,47,50,51,52].

The distribution of ESBL-associated and other resistance determinants in the present study indicates differential selective pressures across wastewater and food animal production environments. The predominance of *CTX-M*-type enzymes, particularly *bla_CTX-M_* variants, is consistent with the global expansion of these ESBLs as dominant drivers of β-lactam resistance in *Klebsiella* and other Enterobacterales [43,44,45]. The concurrent detection of AmpC β-lactamase genes alongside ESBL determinants suggests the co-existence of multiple enzymatic pathways contributing to reduced β-lactam susceptibility within the same ecological settings [53,54]. The presence of plasmid-mediated quinolone resistance genes together with mutations in *gyrA* observed in our study indicates that both horizontal gene transfer and chromosomal mechanisms may be contributing to fluoroquinolone resistance in these populations [55,56]. The higher frequency of resistance determinants in poultry-associated samples relative to cattle farms suggests that poultry production systems may represent more intense or heterogeneous reservoirs for AMR selection and maintenance, potentially reflecting differences in antimicrobial usage patterns, animal density, or environmental contamination pathways [11,23,57,58]. Similarly, the relatively high detection of *bla_CTX-M-1_* across municipal wastewater isolates indicates that wastewater systems may act as convergence points for diverse resistance determinants originating from human, agricultural, and environmental inputs [16,49,52]. These patterns highlight the interconnected nature of resistance ecology across wastewater and food production systems and underscore the importance of integrated One Health surveillance strategies to monitor and mitigate dissemination risks [6,11,23,59].

The diverse AMR gene repertoire identified among the sequenced *K. pneumoniae* isolates in the current study highlights the role of wastewater and food animal production environments as reservoirs of clinically important resistance determinants and is consistent with the literature [49,50]. The detection of genes conferring resistance to β-lactams, aminoglycosides, fluoroquinolones, folate pathway inhibitors, tetracyclines, phenicols, and fosfomycin indicates the accumulation of resistance mechanisms against multiple antimicrobial classes, consistent with the multidrug-resistant phenotypes observed in this study [52,60,61,62]. Of particular concern was the presence of ESBL, AmpC β-lactamase, and carbapenemase genes, as these enzymes substantially reduce the effectiveness of critically important antimicrobials used to treat invasive Gram-negative infections [43,45,53]. The identification of plasmid-mediated quinolone resistance genes and *mcr* genes further underscores the potential for environmental reservoirs to maintain determinants associated with reduced susceptibility to last-line therapeutic agents [55,63]. Our results suggest that municipal wastewater and food animal production systems may contribute to the persistence and circulation of AMR genes within the broader One Health continuum.

The identification of AMR genes, virulence-associated genes, and MGEs within the same isolates in this study underscores the genomic complexity of ESBL-*K. pneumoniae* circulating in municipal wastewater and food animal production environments. Although only six plasmid replicons were found to harbor AMR genes, the detection of *bla_CTX-M-1_*, *qnrS2*, *qnrB19* and *aph(6)-ld* on IncI1, IncQ2, Col440I and IncFII(pECLA) plasmids, respectively, highlights the role of plasmids in maintaining and disseminating resistance determinants [49,64,65,66]. The co-occurrence of virulence-associated genes (*traT*, *hlyF*, *ompT, anr*, and *clpK1*) on IncF-family plasmids further suggests that these plasmids may enhance bacterial fitness, persistence, and pathogenic potential [48,67,68]. The presence of insertion sequences, including ISEc9, ISEc31, and IS609, alongside resistance and virulence genes is noteworthy, as these MGEs can facilitate genomic rearrangements and the mobilization of adaptive traits [68,69]. In particular, the co-localization of IS609 with *hlyE* on the same contig is consistent with the possibility that mobile elements contribute to the dissemination of virulence-associated genes, although functional studies are required to confirm this mechanism [36,48,68,69]. Collectively, these findings highlight the importance of wastewater and food animal production environments as reservoirs of MGEs and plasmids carrying clinically relevant determinants of high-risk *K. pneumoniae* lineages [48,68].

The core genome SNP analysis in this present study revealed substantial genomic diversity among ESBL-*K. pneumoniae* isolates, indicating the circulation of multiple genetic lineages across municipal wastewater and food animal production environments. The identification of genetically indistinguishable isolates (0 core genome SNPs) across wastewater and farm environments provides strong evidence of the circulation of closely related *Klebsiella* strains between environmental and agricultural settings [70,71]. Such minimal genetic distances are consistent with recent common ancestry or transmission events and support the hypothesis that wastewater systems and livestock production environments are interconnected reservoirs for antimicrobial-resistant bacteria [70,71]. The shared AMR genes and virulence genes among these isolates further support their genomic similarity, and this observation is consistent with the literature [51,72]. The close relationship between isolates recovered from duck and cattle drinking water further suggests that shared water sources or environmental contamination may facilitate bacterial dissemination within farms [73,74,75]. Likewise, the detection of only six SNP differences between a chicken drinking water isolate and a cattle fecal isolate indicates recent evolutionary divergence, reinforcing the likelihood of cross-species or environmental transmission [11,23,73,74]. Although these findings do not establish the direction or timing of dissemination, they are consistent with a recent common ancestor or a shared environmental source and are in agreement with previous studies reporting closely related *K. pneumoniae* isolates [50,51,76].

Similar observations have been reported in previous One Health studies, where closely related *K. pneumoniae* isolates have been identified across environmental, animal, and human settings, underscoring the importance of wastewater and agricultural environments as potential reservoirs of antimicrobial-resistant lineages [27,51,77]. Continued genomic surveillance integrating epidemiological and environmental data is needed to better understand the transmission dynamics and persistence of ESBL-*K. pneumoniae* within interconnected One Health systems [11,23,59].

Together, these results emphasize wastewater and agricultural systems as important ecological niches where AMR determinants may be sustained and redistributed. Our study therefore contributes to growing evidence that environmental interfaces linked to human and animal activity play a critical role in the broader epidemiology of ESBL-producing Enterobacterales and reinforces the need for integrated genomic surveillance across interconnected One Health settings.

This study has several limitations. The number of sampled farms and wastewater treatment plants was limited, which may affect the generalizability of the findings. Differences in sampling frequency and sample availability across sites may also have introduced variability in isolate recovery. In addition, sampling was restricted to a single season (May–September 2025), which does not allow assessment of potential seasonal variation. Only a subset of isolates was subjected to WGS due to resource constraints, which may limit the resolution of genomic analyses across the full collection. Antibiotic residue levels were not assessed, preventing evaluation of environmental selection pressures. Despite these limitations, the study provides useful baseline data on the occurrence and genomic characteristics of ESBL-producing *Klebsiella pneumoniae* in wastewater and food animal production environments.

## 5. Conclusions

This study demonstrates that municipal wastewater treatment plants and food animal production environments in southeastern North Carolina harbor genetically diverse ESBL-producing *K. pneumoniae* carrying clinically important AMR determinants, virulence-associated genes, plasmid replicons and MGEs. The detection of closely related isolates across wastewater and agricultural settings highlights the ecological interconnectedness of these environments and underscores their role in the maintenance of antimicrobial-resistant *K. pneumoniae* within a One Health context.

Future studies should incorporate larger numbers of sampling sites and longitudinal sampling to better capture temporal and spatial variation in ESBL-producing populations. Expanded whole-genome sequencing of all isolates, coupled with environmental measurements such as antimicrobial residue quantification, would improve understanding of selection pressures and resistance dynamics. In addition, integration of human clinical isolates and advanced genomic epidemiology approaches would help clarify transmission pathways and better define the role of wastewater and food animal systems in the dissemination of high-risk *K. pneumoniae* lineages.

## Figures and Tables

**Table 1:** Primers used for multiplex and singleplex PCR detection of antimicrobial resistance genes in *Klebsiella pneumoniae* isolates.

Resistance Genes	Forward–Primers DNA Sequence (5′ to 3′)	Reverse–Primers DNA Sequence (5′ to 3′)	Amplicon Sizes (bp)	References
Multiplex PCR assay A				
*bla_CTX-M-1_*	CCGTTTCCGCTATTACAAACCG	GGCCCATGGTTAAAAAATCACTGC	941	[29]
*bla_SHV_*	ATGCGTTATATTCGCCTGTG	AGATAAATCACCACAATGCGC	896	
Multiplex PCR assay B				
*bla_ACT_*	ATTCGTATGCTGGATCTCGCCACC	CATGACCCAGTTCGCCATATCCTG	396	[29]
*bla_FOX_*	CACCACGAGAATAACC	GCCTTGAACTCGACCG	1184	
Multiplex PCR assay C				
*aadA1*	ATGAGGGAAGCGGTGATCG	TTATTTGCCGACTACCTTGGTG	792	[29]
*aphA6*	ATGGAATTGCCCAATATTATTC	TCAATTCAATTCATCAAGTTTTA	781	
Multiplex PCR assay D				
*qnrB*	GATCGTGAAAGCCAGAAAGG	ACGATGCCTGGTAGTTGTCC	469	[29]
*qnrS*	ACGACATTCGTCAACTGCAA	TAAATTGGCACCCTGTAGGC	417	
*gyrA*	CGACCTTGCGAGAGAAAT	GTTCCATCAGCCCTTCAA	626	
Singleplex PCR				
*bla_CTX-M-14_*	GAGAGTGCAACGGATGATG	TGCGGCTGGGTAAAATAG	944	[29]

**Table 2:** Antimicrobial resistance profiles of ESBL-*Klebsiella* isolates from food production environments and municipal wastewater treatment plants in Fayetteville, NC 2025.

Drug Class	Antimicrobial Drugs	Resistance Breakpoint (mm)	Total (*n* = 68; %)	Cattle Farm (*n* = 2; %)	Poultry Farm (*n* = 15; %)	WWTP-A (*n* = 26; %)	WWTP-B (*n* = 25; %)
Tetracycline	Tetracycline (30 µg)	≤11	31 (45.6)	2 (100.0)	9 (60.0)	11 (42.3)	19 (76.0)
Folate pathway antagonists	Trimethoprim/Sulfamethoxazole (5 µg/300 µg)	≤15	36 (52.9)	2 (100.0)	10 (66.7)	14 (53.8)	10 (40.0)
Penicillins	Ampicillin (10 µg)	≤13	57 (83.8)	2 (100.0)	14 (93.3)	22 (84.6)	19 (76.0)
Quinolones	Ciprofloxacin (5 µg)	≤21	39 (57.4)	1 (50.0)	4 (26.7)	16 (61.5)	18 (72.0)
Aminoglycosides	Gentamicin (10 µg)	≤14	14 (20.6)	1 (50.0)	3 (20.0)	4 (15.4)	6 (24.0)
Phenicols	Chloramphenicol (30 µg)	≤12	27 (39.7)	2 (100.0)	8 (53.3)	9 (34.6)	8 (32.0)
Penems	Imipenem (10 µg)	≤19	16 (23.5)	0 (0.0)	1 (6.7)	9 (34.6)	6 (24.0)
Cephalosporins	Cefoxitin (30 µg)	≤14	36 (52.9)	2 (100.0)	10 (66.7)	16 (61.5)	8 (32.0)

**Table 3:** Characteristics of carbapenemase gene-positive ESBL-producing *Klebsiella* isolates identified by WGS.

Isolate ID	Location	Sample Type	Carbapenamase Genes Detected	Imipenem Phenotype	Included in WGS
CCIG1KBE	WWTP-A	Influent	*cphA6*	Resistant	Yes
RFSG1KBE	WWTP-B	Sludge	*blaKPC-2*	Resistant	Yes
22T1KBE	Cattle farm	Feces	*cphA4*	Susceptible	Yes
27T1KB	Cattle farm	Water	*cphA4*	Susceptible	Yes

**Table 4:** Distribution of plasmid replicons, antimicrobial resistance (AMR) genes, virulence factors, and mobile genetic elements in *K. pneumoniae* isolates.

Isolate ID	Source	Plasmid Carrying AMR Genes	AMR Genes	Other MGEs Detected	Virulence Gene	Contig Number
41T3KBE	Poultry farm	IncI1	*bla_CTX-M-1_*	ISEc9		9
48T7KBE	Poultry farm	IncI1	*qacE + sul*	ISEc58		78
		IncFIC(FII)		IS100 + ISEc32	*traT* + *anr*	50
			*aadA1 + bla_CMY-2_*	ISEc9	*cib*	45
		IncFIB(AP001918)			*ompT + hlyF*	79
5T3KBE	WWTP-A	RepA	*aph(3”)-lb + aph(3”)-lb*	IS5 + 15Ppu12 + ISKox3 + ISKpn26 + IS6100		12
		IncFII(pECLA)	*aph(6)-ld*	ISKpn42		17
			*bla_CTX-M-1_*	ISEc9		31
RFSG1KBE	WWTP-B	IncQ2	*qnrS2*			423
CCSG1KBE	WWTP-A	Col440I	*qnrB1*			49
49T1KBE	Poultry farm	IncI1	*bla_CTX-M-1_*			45
		InFII			*traT* + *anr*	76
		IncFIB(AP001918)			*ompT + hlyF*	138

## Data Availability

The whole-genome sequencing data generated in this study have been deposited in the National Center for Biotechnology Information (NCBI) under BioProject accession number PRJNA1092662. Individual Sequence Read Archive (SRA) and BioSample accession numbers are provided in Supplementary Table S1. All other data supporting the findings of this study are included within the article and its Supplementary Materials. Additional data are available from the corresponding author upon reasonable request.

## Supplementary Materials

The following supporting information can be downloaded at: https://www.mdpi.com/article/10.3390/pathogens15080820/s1, Table S1: Metadata and accession numbers for whole-genome-sequenced *Klebsiella pneumoniae* isolates.
