# Supplementary material for: Genomic Surveillance of ESBL-Producing Klebsiella pneumoniae Across Municipal Wastewater and Food Animal Production Environments
Source: Pathogens. 2026 Aug 4;15(8):820. doi: 10.3390/pathogens15080820 (PMC13516603; doi:10.3390/pathogens15080820)
Supplement: Supplementary file 1 [file pathogens-15-00820-s001.zip › pathogens-4443040-supplementary.pdf]

Supplementary Table S1: Metadata and accession numbers for whole-genome-sequenced *Klebsiella pneumoniae* isolates

SRA Accession	Biosample Accession	Strain	Sample_name	bioproject_accession
SRR35888738	SAMN52931349	CCIG1KBE	CCIG1KBE	PRJNA1092662
SRR35888741	SAMN52931350	CCEG1KB	CCEG1KB	PRJNA1092662
SRR35888739	SAMN52931351	CCIC1KBE	CCIC1KBE	PRJNA1092662
SRR35888740	SAMN52931352	CCEG2KB	CCEG2KB	PRJNA1092662
SRR35888758	SAMN52931353	RFSG1KBE	RFSG1KBE	PRJNA1092662
SRR35888761	SAMN52931354	RFEG2KB	RFEG2KB	PRJNA1092662
SRR35888762	SAMN52931355	CCSG1KBE	CCSG1KBE	PRJNA1092662
SRR35888760	SAMN52931356	RFIC1KBE	RFIC1KBE	PRJNA1092662
SRR35888759	SAMN52931357	RFIG2KBE	RFIG2KBE	PRJNA1092662
SRR35888737	SAMN52931358	CCSG1KB	CCSG1KB	PRJNA1092662
SRR35888347	SAMN52930672	7T1KB	7T1KB	PRJNA1092662
SRR35888355	SAMN52930673	8T1KBE	8T1KBE	PRJNA1092662
SRR35888356	SAMN52930674	9T1KB	9T1KB	PRJNA1092662
SRR35888348	SAMN52930675	10T1KBE	10T1KBE	PRJNA1092662
SRR35888349	SAMN52930676	11T1KB	11T1KB	PRJNA1092662
SRR35888350	SAMN52930677	15T1KB	15T1KB	PRJNA1092662
SRR35888351	SAMN52930678	16T1KB	16T1KB	PRJNA1092662
SRR35888352	SAMN52930679	18T1KB	18T1KB	PRJNA1092662
SRR35888353	SAMN52930680	22T1KBE	22T1KBE	PRJNA1092662
SRR35888354	SAMN52930681	25T1KB	25T1KB	PRJNA1092662
SRR35888340	SAMN52930682	26T1KB	26T1KB	PRJNA1092662
SRR35888341	SAMN52930683	27T1KB	27T1KB	PRJNA1092662
SRR35888342	SAMN52930684	28T1KB	28T1KB	PRJNA1092662
SRR35888343	SAMN52930685	3T1KB	3T1KB	PRJNA1092662
SRR35888345	SAMN52930686	49T1KBE	49T1KBE	PRJNA1092662
SRR35888344	SAMN52930687	49T1KB	49T1KB	PRJNA1092662
SRR35888346	SAMN52930688	51T1KB	51T1KB	PRJNA1092662
Yet to be assigned		01T1KBE	01T1KBE	PRJNA1092662
Yet to be assigned		02T1KBE	02T1KBE	PRJNA1092662
Yet to be assigned		11T3KBE	11T3KBE	PRJNA1092662
Yet to be assigned		2T3KBE	2T3KBE	PRJNA1092662
Yet to be assigned		40T3KBE	40T3KBE	PRJNA1092662
Yet to be assigned		41T3KBE	41T3KBE	PRJNA1092662
Yet to be assigned		53T3KBE	53T3KBE	PRJNA1092662
Yet to be assigned		5T3KBE	5T3KBE	PRJNA1092662
Yet to be assigned		9T3KBE	9T3KBE	PRJNA1092662
Yet to be assigned		18T7KBE	18T7KBE	PRJNA1092662
Yet to be assigned		46T2KBE	46T2KBE	PRJNA1092662
Yet to be assigned		48T7KBE	48T7KBE	PRJNA1092662
Yet to be assigned		5T2KBE	5T2KBE	PRJNA1092662
Yet to be assigned		6T3KBE	6T3KBE	PRJNA1092662
Yet to be assigned		7T3KBE	7T3KBE	PRJNA1092662
Yet to be assigned		8T3KBE	8T3KBE	PRJNA1092662

Sample_type	Source	Organism	Collected_by
Influent	Wastewater	Klebsiella pneumoniae	Aworh Lab, Fayetteville State University
Effluent	Wastewater	Klebsiella pneumoniae	Aworh Lab, Fayetteville State University
Influent	Wastewater	Klebsiella pneumoniae	Aworh Lab, Fayetteville State University
Effluent	Wastewater	Klebsiella pneumoniae	Aworh Lab, Fayetteville State University
Sludge	Wastewater	Klebsiella pneumoniae	Aworh Lab, Fayetteville State University
Effluent	Wastewater	Klebsiella pneumoniae	Aworh Lab, Fayetteville State University
Sludge	Wastewater	Klebsiella pneumoniae	Aworh Lab, Fayetteville State University
Influent	Wastewater	Klebsiella pneumoniae	Aworh Lab, Fayetteville State University
Influent	Wastewater	Klebsiella pneumoniae	Aworh Lab, Fayetteville State University
Sludge	Wastewater	Klebsiella pneumoniae	Aworh Lab, Fayetteville State University
Influent	Wastewater	Klebsiella pneumoniae	Aworh Lab, Fayetteville State University
Influent	Wastewater	Klebsiella pneumoniae	Aworh Lab, Fayetteville State University
Sludge	Wastewater	Klebsiella pneumoniae	Aworh Lab, Fayetteville State University
Sludge	Wastewater	Klebsiella pneumoniae	Aworh Lab, Fayetteville State University
Sludge	Wastewater	Klebsiella pneumoniae	Aworh Lab, Fayetteville State University
Effluent	Wastewater	Klebsiella pneumoniae	Aworh Lab, Fayetteville State University
Effluent	Wastewater	Klebsiella pneumoniae	Aworh Lab, Fayetteville State University
Effluent	Wastewater	Klebsiella pneumoniae	Aworh Lab, Fayetteville State University
Cow Feces	Cattle Farm	Klebsiella pneumoniae	Aworh Lab, Fayetteville State University
Cow Drinking water	Cattle Farm	Klebsiella pneumoniae	Aworh Lab, Fayetteville State University
Cow Drinking water	Cattle Farm	Klebsiella pneumoniae	Aworh Lab, Fayetteville State University
Cow Drinking water	Cattle Farm	Klebsiella pneumoniae	Aworh Lab, Fayetteville State University
Cow Drinking water	Cattle Farm	Klebsiella pneumoniae	Aworh Lab, Fayetteville State University
Effluent	Wastewater	Klebsiella pneumoniae	Aworh Lab, Fayetteville State University
Duck Drinking Water	Poultry Farm	Klebsiella pneumoniae	Aworh Lab, Fayetteville State University
Duck Drinking Water	Poultry Farm	Klebsiella pneumoniae	Aworh Lab, Fayetteville State University
Duck Drinking Water	Poultry Farm	Klebsiella pneumoniae	Aworh Lab, Fayetteville State University
Influent	Wastewater	Klebsiella pneumoniae	Aworh Lab, Fayetteville State University
Influent	Wastewater	Klebsiella pneumoniae	Aworh Lab, Fayetteville State University
Sludge	Wastewater	Klebsiella pneumoniae	Aworh Lab, Fayetteville State University
Influent	Wastewater	Klebsiella pneumoniae	Aworh Lab, Fayetteville State University
Chicken Feces	Poultry Farm	Klebsiella pneumoniae	Aworh Lab, Fayetteville State University
Chicken Feces	Poultry Farm	Klebsiella pneumoniae	Aworh Lab, Fayetteville State University
Effluent	Wastewater	Klebsiella pneumoniae	Aworh Lab, Fayetteville State University
Influent	Wastewater	Klebsiella pneumoniae	Aworh Lab, Fayetteville State University
Sludge	Wastewater	Klebsiella pneumoniae	Aworh Lab, Fayetteville State University
Effluent	Wastewater	Klebsiella pneumoniae	Aworh Lab, Fayetteville State University
Duck Feces	Poultry Farm	Klebsiella pneumoniae	Aworh Lab, Fayetteville State University
Chicken water	Poultry Farm	Klebsiella pneumoniae	Aworh Lab, Fayetteville State University
Influent	Wastewater	Klebsiella pneumoniae	Aworh Lab, Fayetteville State University
Influent	Wastewater	Klebsiella pneumoniae	Aworh Lab, Fayetteville State University
Influent	Wastewater	Klebsiella pneumoniae	Aworh Lab, Fayetteville State University
Influent	Wastewater	Klebsiella pneumoniae	Aworh Lab, Fayetteville State University

Sequenced_by
North Carolina State University|College of Veterinary Medicine|Thakur Molecular Epidemiology Laboratory
North Carolina State University|College of Veterinary Medicine|Thakur Molecular Epidemiology Laboratory
North Carolina State University|College of Veterinary Medicine|Thakur Molecular Epidemiology Laboratory
North Carolina State University|College of Veterinary Medicine|Thakur Molecular Epidemiology Laboratory
North Carolina State University|College of Veterinary Medicine|Thakur Molecular Epidemiology Laboratory
North Carolina State University|College of Veterinary Medicine|Thakur Molecular Epidemiology Laboratory
North Carolina State University|College of Veterinary Medicine|Thakur Molecular Epidemiology Laboratory
North Carolina State University|College of Veterinary Medicine|Thakur Molecular Epidemiology Laboratory
North Carolina State University|College of Veterinary Medicine|Thakur Molecular Epidemiology Laboratory
North Carolina State University|College of Veterinary Medicine|Thakur Molecular Epidemiology Laboratory
North Carolina State University|College of Veterinary Medicine|Thakur Molecular Epidemiology Laboratory
North Carolina State University|College of Veterinary Medicine|Thakur Molecular Epidemiology Laboratory
North Carolina State University|College of Veterinary Medicine|Thakur Molecular Epidemiology Laboratory
North Carolina State University|College of Veterinary Medicine|Thakur Molecular Epidemiology Laboratory
North Carolina State University|College of Veterinary Medicine|Thakur Molecular Epidemiology Laboratory
North Carolina State University|College of Veterinary Medicine|Thakur Molecular Epidemiology Laboratory
North Carolina State University|College of Veterinary Medicine|Thakur Molecular Epidemiology Laboratory
North Carolina State University|College of Veterinary Medicine|Thakur Molecular Epidemiology Laboratory
North Carolina State University|College of Veterinary Medicine|Thakur Molecular Epidemiology Laboratory
North Carolina State University|College of Veterinary Medicine|Thakur Molecular Epidemiology Laboratory
North Carolina State University|College of Veterinary Medicine|Thakur Molecular Epidemiology Laboratory
North Carolina State University|College of Veterinary Medicine|Thakur Molecular Epidemiology Laboratory
North Carolina State University|College of Veterinary Medicine|Thakur Molecular Epidemiology Laboratory
North Carolina State University|College of Veterinary Medicine|Thakur Molecular Epidemiology Laboratory
North Carolina State University|College of Veterinary Medicine|Thakur Molecular Epidemiology Laboratory
North Carolina State University|College of Veterinary Medicine|Thakur Molecular Epidemiology Laboratory
North Carolina State University|College of Veterinary Medicine|Thakur Molecular Epidemiology Laboratory
North Carolina State University|College of Veterinary Medicine|Thakur Molecular Epidemiology Laboratory
North Carolina State University|College of Veterinary Medicine|Thakur Molecular Epidemiology Laboratory
North Carolina State University|College of Veterinary Medicine|Thakur Molecular Epidemiology Laboratory
North Carolina State University|College of Veterinary Medicine|Thakur Molecular Epidemiology Laboratory
North Carolina State University|College of Veterinary Medicine|Thakur Molecular Epidemiology Laboratory
North Carolina State University|College of Veterinary Medicine|Thakur Molecular Epidemiology Laboratory
North Carolina State University|College of Veterinary Medicine|Thakur Molecular Epidemiology Laboratory
North Carolina State University|College of Veterinary Medicine|Thakur Molecular Epidemiology Laboratory
North Carolina State University|College of Veterinary Medicine|Thakur Molecular Epidemiology Laboratory
North Carolina State University|College of Veterinary Medicine|Thakur Molecular Epidemiology Laboratory
North Carolina State University|College of Veterinary Medicine|Thakur Molecular Epidemiology Laboratory
North Carolina State University|College of Veterinary Medicine|Thakur Molecular Epidemiology Laboratory
North Carolina State University|College of Veterinary Medicine|Thakur Molecular Epidemiology Laboratory
North Carolina State University|College of Veterinary Medicine|Thakur Molecular Epidemiology Laboratory
North Carolina State University|College of Veterinary Medicine|Thakur Molecular Epidemiology Laboratory
North Carolina State University|College of Veterinary Medicine|Thakur Molecular Epidemiology Laboratory

Collection_year	Location	
2025	USA: NC	
2025	USA: NC	
2025	USA: NC	
2025	USA: NC	
2025	USA: NC	
2025	USA: NC	
2025	USA: NC	
2025	USA: NC	
2025	USA: NC	
2025	USA: NC	
2025	USA: NC	
2025	USA: NC	
2025	USA: NC	
2025	USA: NC	
2025	USA: NC	
2025	USA: NC	
2025	USA: NC	
2025	USA: NC	
2025	USA: NC	
2025	USA: NC	
2025	USA: NC	
2025	USA: NC	
2025	USA: NC	
2025	USA: NC	
2025	USA: NC	
2025	USA: NC	
2025	USA: NC	
2025	USA: NC	
2025	USA: NC	
2025	USA: NC	
2025	USA: NC	
2025	USA: NC	
2025	USA: NC	
2025	USA: NC	
2025	USA: NC	
2025	USA: NC	
2025	USA: NC	
2025	USA: NC	
2025	USA: NC	
2025	USA: NC	
2025	USA: NC	
2025	USA: NC	
2025	USA: NC	
